# Factors related to serum levels of intercellular adhesion molecule-1 in probable COVID-19 patients in surgical treatment: an observational study

**DOI:** 10.11604/pamj.2023.46.117.41690

**Published:** 2023-12-27

**Authors:** Mendy Hatibie Oley, Maximillian Christian Oley, Fima Lanra Fredrik Gerald Langi, Billy Johnson Kepel, Jacob Pajan, Regina Elizabeth Meriam Kepel, Angelica Maurene Joicetine Wagiu, Ferry Kalitouw, Laurens Kalesaran, Muhammad Faruk

**Affiliations:** 1 Division of Plastic Reconstructive and Aesthetic Surgery, Department of Surgery, Faculty of Medicine, Sam Ratulangi University, Manado, Indonesia,; 2 Division of Plastic Reconstructive and Aesthetic Surgery, Department of Surgery, R. D. Kandou Hospital, Manado, Indonesia,; 3 Division of Neurosurgery, Department of Surgery, Faculty of Medicine, Sam Ratulangi University, Manado, Indonesia,; 4 Division of Neurosurgery, Department of Surgery, R. D. Kandou Hospital, Manado, Indonesia,; 5 Department Epidemiology and Biostatistics, Public Health Faculty, Sam Ratulangi University, Manado, Indonesia,; 6 Department of Chemistry, Faculty of Medicine, Sam Ratulangi University, Manado, North Sulawesi, Indonesia,; 7 Department of Surgery, Faculty of Medicine, Sam Ratulangi University, Manado, Indonesia,; 8 North Sulawesi Regional Hospital, Manado, Indonesia,; 9 Department of Surgery, Faculty of Medicine, Hasanuddin University, Makassar, Indonesia

**Keywords:** Intercellular Adhesion Molecule 1, COVID-19, surgery, patients

## Abstract

**Introduction:**

COVID-19 causes a systemic inflammatory response, involving dysregulation and misexpression of many inflammatory cytokines. The recruitment and activation of inflammatory cells depend on the expression of many classes of inflammatory mediators, with increased expression of endothelial cell adhesion molecules being related to COVID-19 disease severity. With the World Health Organization having recently updated case definitions to suspect, probable, and confirmed, this study aimed to measure the mean value of intercellular adhesion molecule 1 (ICAM-1) and its relation to suspected COVID-19.

**Methods:**

all suspected patients (n=20) were hospitalized and treated following the Indonesian National Guidelines for COVID-19 management. ICAM-1 levels were measured on days 1 and 7, demographic data were recorded, and routine blood count values were measured and additionally considered.

**Results:**

the results showed that the levels of ICAM-1 in the 1^st^-day group (mean 271.3 ng/ml) were higher than those in the 7^th^-day group (mean 253.9 ng/ml). This difference was statistically significant (p = 0.00, p ≤ 0.05). All of the patients with suspected COVID-19 were included in this study and tested for COVID-19 by reverse transcription polymerase chain reaction (RT-PCR) testing. A total of 10 patients were confirmed positive with a COVID-19 infection, with elevated ICAM-1 levels compared to the confirmed negative patients (with a mean 1^st^ day 296.8 versus a mean 7th day 279.0 ng/ml). ICAM-1 levels of all patients decreased by the seventh day.

**Conclusion:**

the mean value of ICAM-1 levels for patients with confirmed positive COVID-19 cases was higher than those with suspected COVID-19 cases.

## Introduction

SARS-CoV-2 (COVID-19) emerged in early December 2019, resulting in a global pandemic [1-3]. Data showed that, as of September 2, 2020, more than 25 million people had been confirmed as positive COVID-19 cases worldwide, which presented an overall mortality rate of over 3.3% [4]. In the course of the disease, COVID-19 can cause pneumonia and Acute respiratory distress syndrome (ARDS), as well as damage to other organs and systems, including cerebrovascular diseases such as strokes [5-7]. The COVID-19 pandemic motivated researchers to develop convenient and practical methods to allow doctors to quickly identify high-risk patients. Identification and measurement of risk factor parameters for intervention and early management are needed to support clinical decision-making. COVID-19 causes a systemic inflammatory response, which involves the dysregulation and incorrect expression of many inflammatory cytokines. Inflammatory cell recruitment and activation are dependent on the expression of many classes of inflammatory mediators, such as cytokines (interleukin [IL]-1, IL-6, and IL-18), chemokines (fractalkine [FKN]), and adhesion molecules (Intercellular Adhesion Molecule-1 [ICAM-1]) and Vascular Cell Adhesion Molecule-1 [VCAM-1] [8].

ICAM-1, also known as CD54 (Cluster of Differentiation 54), is a protein that is encoded by the ICAM-1 gene in humans. This gene encodes a cell surface glycoprotein that is normally expressed on endothelial and immune system cells [9-11]. The protein binds to integrins of the CD11a/CD18 or CD11b/CD18 types and is also exploited by rhinoviruses as receptors for entry into the respiratory epithelium [12]. Due to the association with the immune response, it has been hypothesized that ICAM-1 may function in signal transduction. In a retrospective study of 39 COVID-19 patients and 32 control patients in China [8], clinical data were collected to examine the expression of endothelial cell adhesion molecules by the enzyme-associated immunosorbent assay. Serum levels of fractalkines, the molecules VCAM-1, ICAM-1, and Vascular Adhesion Protein-1 (VAP-1) were found to be elevated in patients with mild disease, increase dramatically in severe cases, and decrease in the convalescent phase. In this study, we analyzed ICAM-1 as an endothelial inflammatory adhesion molecule as a biomarker that could be used as a prognostic factor for surgical patient outcomes and determined its relationship to COVID-19 and other related factors.

## Methods

**Study design:** this study was an analytical observational study. The collection of serum ICAM-1 data was carried out before the procedure and immediately until about one week after. Due to the nature of the data collection, the investigation was designed as a prospective cohort study. Surgical treatment was performed on each patient according to the diagnosis and was a routine procedure without any experimental treatment.

**Setting:** data collection and processing were carried out at the Department of Surgery at R.D. Kandou Hospital, Manado, Indonesia.

**Participants:** sample selection and data collection took place from early March to late June 2020. The population of concern for the study were patients with suspected and probable COVID-19 who were treated in the surgical department and had subsequent swab examinations to confirm a COVID-19 infection. Patients treated at the hospital were the target population, whilst the accessible population was the individuals who were included in the target population and treated throughout the data collection period.

### Variables

**ICAM-1:** the value of intercellular adhesion molecule 1 (ICAM-1) in peripheral blood according to the results of immunoassay examination in an accredited laboratory. Expressed in units of ng/mL.

**COVID-19:** the results of the throat swab examination of patients with suspected COVID-19 cases using the polymerase chain reaction method in an accredited laboratory, were declared positive vs. negative.

**Time:** ICAM-1 sampling time from patients: initial, continued.

**Age:** the patient's age at the time of hospital admission, in years.

**Data source/measurement:** the patients underwent blood laboratory examination (complete blood counts, electrolyte, renal function and liver function test) and characteristics data was collected, including serum ICAM-1 and COVID-19 levels before surgery. ICAM-1 levels were measured again one week after the surgical procedure. Peripheral vein blood sampling was performed before and immediately after surgery. In the ICAM-1 examination with Merck Millipore ELISA kit (catalog no: ECM335, Burlington, Massachusetts, USA), blood samples were stored at 2-5oC before being centrifuged at 1000 RPM/min for 60 minutes until coagulation occurred. The results were then stored at -80°C until the time of blood analysis. The whole procedure of further examination and processing of the blood sample is carried out at our institution. The throat swab was immediately sent to the laboratory in a protected container for a Polymerase Chain Reaction (PCR) test for SARS-CoV-2 virus infection using an Abbott RealTime SARS-CoV-2 Assay (Abbott Molecular Inc, Salt Lake City, IL, USA) according to the manufacturer´s instructions.

**Bias:** two of the authors conducted the ICAM-1 and Polymerase Chain Reaction (PCR) test for SARS-CoV-2 virus quantification without access to clinical or analytical data of the patients in order to prevent information and selection bias.

**Study size:** the sample was taken from the population who were treated at the hospital and met the criteria after they gave their consent. The sample size for this study was 39 participants according to the Borm *et al*. formula [13,14].

**Inclusion and exclusion criteria:** patients in the accessible population had the following inclusion criteria; patients are triage patients in the emergency department for surgical procedures; patients meet the suspect and probable COVID-19 criteria; patients are willing to undergo a swab examination for RT-PCR COVID-19; patients give informed consent in writing for this study. Patients were then excluded from the study if they met one or more of the following criteria: the patient refuses to undergo a swab test or be treated; patients with obstetric and gynecological care, including delivery management; the patient dies during treatment.s

**Quantitative variables:** quantitative variables such as age, sex, complete blood counts, electrolyte, renal function and liver function test.

**Statistical methods:** descriptive tabulation of patient characteristics was carried out according to type, namely mean and standard deviation (or if not normally distributed, median and interquartile range [IQR]) on numerical variables and proportions for categorical variables. A description of the patient by gender also includes the results of the t-test (or Mann-Whitney U test) and 2 tests (or Fisher's Exact). Previously, in the preliminary analysis, the univariate distribution of each variable was evaluated using graphs including histograms, boxplots, and Q-Q plots for numeric variables, or bar charts for categorical variables. Determination of the normality of the distribution of the numerical variables was carried out using the Shapiro-Wilk test. Factors associated with serum ICAM-1 levels were identified using linear regression analysis. The results of the regression analysis are presented as the estimated values of the regression parameters and the 95% confidence interval, as well as their p-values. Data processing and statistical analyses were carried out using R Statistical Software (version 4.0.1) [15]. Descriptive tabulation, graphing, and regression modeling are all based on routine packages in the software. Preparation of data from raw form to a format that is ready for analysis was completed using Microsoft Excel 2017.

**Ethical consideration:** this study was implemented after the principal investigator obtained ethical clearance from our Research Ethics Commission (with number PP04.03/XIX.2/864/2020) and has been registered with the Thai Clinical Trials Registry (no. TCTR20220919005).

## Results

**Participants:** a total of 40 surgical patients with variant diagnoses were included in the study data (Table 1).

**Table 1:** characteristics of patients included in the study

Variable	Total (N = 40)		Male (n = 26)		Female (n = 14)		p-value^a^
	Mean± SD	Median (Q_1_; Q_3_)	Mean±SD	Median (Q_1_; Q_3_)	Mean ±SD	Median (Q_1_; Q_3_)	
Age (years)		53.0 (42.8; 60,2)		53.0 (42.2; 60.0)		51.5 (46.0; 63.0)	1.000
Hemoglobin (g/dL)	10.7 ±2.7		11.0 ±3.2		10.1±1.3		0.222
Leukocytes (×10^3^/µL)		14.9 (9.0; 21.2)		12.8 (8.2; 21.3)		16.9 (13.3; 21.0)	0.192
Erythrocytes (×10^6^/µL)	3.8 ±1.0		3.9 ±1.1		3.7 ±0.6		0.480
Hematocryte (%)	31.4 ±7.8		32.2 ±9.1		29.9 ±4.3		0.282
Platelets (×10 ^3^/µL)		269.0 (215.5; 450.5)		262.0 (214.5; 446.2)		372.0 (268.2; 439.2)	0.173
Eosinofil (%)		0.0 (0.0; 2.0)		0.0 (0.0; 2.2)		1.0 (0.0; 2.0)	0.680
Rod Neutrophils (%)		2.5 (0.0; 9.0)		2.0 (0.0; 13.0)		3.0 (1.0; 4.5)	0.574
Segmented Neutrophils (%)		70.0 (61.0; 78.0)		67.5 (56.8; 76.5)		71.0 (67.0; 80.0)	0.161
Lymphocytes (%)	16.9 ±7.9		17.0 ±8.1		16.5 ±7.7		0.856
Monocytes (%)		6.0 (4.0; 9.0)		6.0 (4.0; 8.2)		6.0 (4.0; 9.0)	0.872
NLR		4.0 (2.6; 5.8)		4.4 (2.4; 5.6)		3.5 (3.2; 7.7)	0.656
Sodium (mg/dL)	133.9 ±8.0		134.7 ±8.7		132.8 ±6.9		0.504
Potassium (mg/dL)		4.2 (3.5; 4.7)		4.2 (4.0; 4.8)		4.2 (3.4; 4.6)	0.478
Chloride (mg/dL)	96.2 ±8.9		98.1 ±8.3		93.4 ±9.4		0.141
SGOT (mg/dL)		27.5 (18.2; 46.2)		23.0 (17.0; 47.0)		35.0 (23.0; 44.0)	0.435
SGPT (mg/dL)		19.0 (13.0; 25.2)		19.0 (14.0; 36.0)		18.0 (13.0; 20.0	0.279
Urea (mg/dL)		38.0 (24.0; 97.0)		38.5 (24.2; 82.2)		31.0 (24.0; 149.0)	0,864
Creatinine (mg/dL)		1.1 (0.7; 2.0)		1.1 (0.8; 2.0)		0.9 (0.6; 1.7)	0.214
COVID-19, n (%)							
Not tested	6 (15)		5 (19)		1 (7)		0.723
Negative	24 (60)		15 (58)		9 (64)		
Positive	10 (25)		6 (23)		4 (29)		
ICAM-1 serum Level (ng/mL)							
Initial		265.4 (182.1; 315.0)		265.4 (193.6; 314.1)		264.0 (155.0; 315.0)	0.769
Continued		236.3 (169.3; 308.4)		229.2 (180.6; 304.6)		262.3 (141.3; 310.9)	0.967
Changed		-0.7 (-8.6; 13.6)		0.5 (-7.7; 18.9)		-2.6 (-15.9; 4.3)	0.361

a t-test or Mann-Whitney U test on numeric variables, 2 tests (or Fisher's Exact) on categorical variables; SD, standard deviation; Q1, Quartile 1; Q3, Quartile 3; SGOT, serum glutamic oxaloacetic transaminase; SGPT, serum glutamate-pyruvate transaminase; NLR, Neutrophil Lymphocyte Ratio

**Descriptive data:** a total of 10 (25%) patients in the study were found to be positive for COVID-19 based on RT-PCR examination, with a male to female ratio of 6: 4. Table 2 showed that the difference in the proportion of COVID-19 test results is not particularly different between the two sexes. The median baseline level of ICAM-1 in the serum of the patients in the study was around 265.4 ng/mL (IQR 182.1-315.0 ng/mL), whilst at follow-up, it was around 236.3 ng/mL (IQR 169.3-mL-308.4 ng/mL). Although the median overall patient score appeared to fall between the two tests, the magnitude of the change in serum ICAM-1 measured for each individual to overcome the problem of statistical dependence on initial and follow-up examinations was very minimal. It should be noted that the IQR did not represent a significant change as the range included values of zero. There were no significant differences in serum ICAM-1 levels according to gender for initial examination, follow-up, or changes between the two tests.

**Table 2:** univariate linear regression model initial levels and changes in serum levels of ICAM-1

Variable	Initial Level		Changes in serum level	
	β (95% CI)	p	β (95% CI)	p
Female vs male	-10.45 (-64.31; 43.41)	0.697	-9.34 (-25.05; 6.36)	0.236
Age	-0.82 ( -2.39; 0.76)	0.301	0.20 ( -0.27; 0.67)	0.394
Hemoglobin	3.92 ( -5.71; 13.54)	0.415	0.27 ( -2.61; 3.15)	0.850
Leukocytes	-1.59 ( -3.71; 0.53)	0.138	-0.58 ( -1.20; 0.04)	0.065
Erythrocytes	9.09 (-17.95; 36.13)	0.500	1.73 ( -6.32; 9.78)	0.666
Platelets	-0.02 ( -0.18; 0.14)	0.778	-0.02 ( -0.07; 0.02)	0.271
Eosinophils	1.21 (-10.27; 12.69)	0.832	-1.50 ( -4.52; 1.51)	0.318
Basophils	29.71 (-50.28; 109.71)	0.456	10.98 (-10.14; 32.11)	0.298
Stabs Neutrophils	1.76 (-1.19; 4.71)	0.233	1.44 (0.88; 2.00)	<0.001
Segmented Neutrophils	-0.55 (-2.19; 1.09)	0.503	-0.70 (-1.07; -0.33)	0.001
Lymphocytes	-1.70 (-5.19; 1.80)	0.332	-0.28 (-1.21; 0.66)	0.556
Monocytes	4.90 (-3.52; 13.32)	0.245	0.27 (-2.02; 2.55)	0.815
NLR	0.61(-5.45; 6.68)	0.838	-0.99 (-2.57; 0.59)	0.212
Sodium	3.37(-0.08; 6.82)	0.056	0.92 (0.01; 1.82)	0.047
Potassium	0.34(-18.60; 19.28)	0.971	0.39 (-4.58; 5.36)	0.874
Chloride	2.28(-0.89; 5.44)	0.153	0.53 (-0.31; 1.37)	0.207
SGOT	0.17(-0.06; 0.41)	0.140	0.01 (-0.05; 0.07)	0.762
SGPT	1.34(-0.28; 2.96)	0.102	0.01 (-0.40; 0.42)	0.965
Urea	-0.09(-0.55; 0.37)	0.699	-0.02 (-0.14; 0.10)	0.722
Creatinine	-2.68(-13.15; 7.80)	0.606	-1.02 (-3.56; 1.53)	0.422
COVID-19	87.94(35.98; 139.90)	0.001	-15.17 (-32.08; 1.74)	0.077

CI, confidence interval; ICAM, intercellular adhesion molecule; SGOT, serum glutamic oxaloacetic transaminase; SGPT, serum glutamate-pyruvate transaminase; NLR, Neutrophil Lymphocyte Ratio

**Figure 1:**
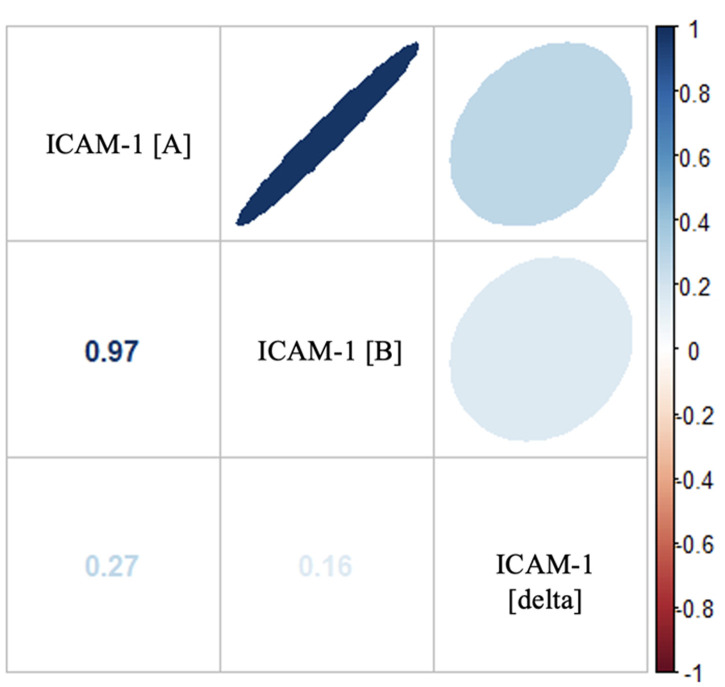
correlation matrix of ICAM-1 serum levels at initial measurement (ICAM1 [A]), follow-up (ICAM1 [B]), and changes in value between the two in each patient (ICAM1 [delta])

**Outcome data:** repeated measurements in each patient caused a natural dependence on the results of the examination of serum ICAM-1 levels. The matrix in Figure 1 demonstrates this dependency, where the correlation between initial and follow-up measurements is r = 0.97 (p < 0.001). The relationship with changes in serum levels was very small and insignificant, r = 0.27 and 0.16 for initial and follow-up examinations, respectively. For this reason, only the results of the initial measurement and the value of the change between the baseline and follow-up measures were used as outcomes for the regression analysis. The variation in elliptical shape expresses the relative size of the relationship between variables, while the quality and intensity of color show the direction and strength of the relationship, where blue means a positive correlation, red means a negative correlation, and a higher color intensity indicates a stronger correlation (Figure 1).

**Main results:** Table 2 shows the results of the regression analysis for the initial serum ICAM-1 levels and their changes between baseline and follow-up examinations, with other variables in Table 2 as predictors. In the first paret, only the presence or absence of COVID-19 was a factor related to serum ICAM-1 levels at baseline. Patients who were positive for COVID-19 based on the results of the RT-PCR test in this study had an average serum ICAM-1 level of about 88 ng/mL (95% CI 36-140 ng/mL, p = 0.001) higher than those who were negative, or not tested. It should be noted that the negative and unchecked categories for the COVID-19 variable were included in the regression analysis to facilitate the interpretation of the results. However, the infection in these patients was not associated with changes in serum ICAM-1 levels. In the second part, it was seen that changes in serum levels of ICAM-1 were only affected by the neutrophil count in both rods and segments. For example, each 1% increase in the rod neutrophil count was, on average, associated with an increase in serum ICAM-1 levels of 1.44 ng/mL (95% CI 0.88-2.00 ng/mL; p ≤0.001) on examination. This trend continued when compared with the patient's baseline serum level. In contrast, a 1% increase in the segment neutrophil count was seen to be associated with a decrease in serum ICAM-1 levels at follow-up by a mean of 0.70 ng/mL (95% CI a decrease of 0.33-1.07 ng/mL; p ≤0.001). No other factors were found in the study data associated with baseline serum ICAM-1 levels or changes between baseline and follow-up examinations.

**Other analyses:** twenty (50%) were treated for diabetic ulcers requiring wound care, 5 (13%) had a history of trauma, 4 (10%) had tumors with obstructive or painful symptoms, and 3 (8%) had peritonitis. 26 (65%) patients were male, with their associated characteristics listed in Table 1. The median age of the patients was 53 years (IQR 43-60 years). On average, patients were anemic with a hemoglobin level of 10.7 (SD 2.7) g/dL. The median leukocyte value for all study subjects tended to be leukocytosis (14.9 thousand/μL, IQR 9.0-21.2 thousand/μL). The appearance of erythrocytes and hematocrit was in accordance with the hemoglobin value, which tends to be below normal. The median rod and segment neutrophils in the differential count were 3% and 70%, respectively. Additionally, the mean lymphocyte value was approximately 16.9% (SD 7.9%), and the median neutrophil-lymphocyte ratio (NLR) was 4.0 (IQR 2.6-5.8). Electrolyte levels, including those for sodium, potassium, and chloride, were within normal limits for all patients. Liver function biomarkers such as serum glutamic-oxaloacetic transaminase (SGOT) and serum glutamic pyruvic transferase (SGPT) median values were also within normal limits. Regarding markers of kidney function, median creatinine levels were near the upper limit of normal values (1.1 mg/dL, IQR 0.7-2.0 mg/dL), whilst urea values were moderately high (38 mg/dL, IQR 24-97 mg/dL). There were no significant differences between the sexes in all of the blood laboratory characteristics mentioned.

## Discussion

The results of this study indicated that variations in measurement can identify changes in the patient's laboratory examination. An increase in leukocytes was seen in almost all patients. As previously described, ICAM-1 is an inflammatory adhesion molecule that then binds and collects leukocytes [16], and this relationship was demonstrated within this research. Another trend observed in this study was that the average level of ICAM-1 was slightly higher but not statistically significant in patients with a confirmed COVID-19 case who underwent surgery. Theoretically, ICAM-1 as a mediator of endothelial inflammation was increased in patients with confirmed COVID-19, because SARS-CoV-2 will trigger an inflammatory cascade in cells through its endothelial receptors, which were then known as cytokine storms [17]. Unfortunately, the number of samples obtained is small; whilst this was a drawback of this study, the author strongly recommended conducting a similar, larger study.

COVID-19 causes a systemic inflammatory response, which involves the dysregulation and incorrect expression of many inflammatory cytokines [18,19]. Inflammatory cell recruitment and activation are dependent on the expression of many classes of inflammatory mediators such as cytokines and adhesion molecules including ICAM-1, vascular cell adhesion molecule-1, pathological venous thromboembolism, direct viral infection of endothelial cells, and endothelial inflammation. These effects were reported in a recent study [17]. Other studies have also found that higher doses of anticoagulation were recommended as therapy [20]. The role of antiphospholipid antibodies, VWF and FVIII, requires further investigation. This study found evidence of the direct viral infection of endothelial cells, as well as diffuse endothelial inflammation. Although the virus uses the angiotensin-converting enzyme 2 (ACE2) receptor expressed by pneumocytes in the alveolar epithelial lining to infect the host, thereby causing lung injury, the ACE2 receptor is also widely expressed on endothelial cells, which traverse many organs [21]. Immune cell recruitment, either by direct viral endothelial infection or immune-mediated, can lead to widespread endothelial dysfunction associated with apoptosis. ICAM-1 is a gene that encodes a cell surface glycoprotein that is normally expressed on endothelial cells and immune system cells. This gene functions in stabilizing cell-cell interactions and facilitating leukocyte endothelial transmigration [22]. Recently, ICAM-1 has been characterized as the cellular entry site of human rhinoviruses. Due to this association with the immune response, it has been hypothesized that ICAM-1 may function in signal transduction. ICAM-1 ligation produces proinflammatory effects, such as recruitment of inflammatory leukocytes, by signaling via a cascade involving several kinases [23].

Based on the demographic characteristics of the patients involved in this study, there were variations in the patterns of those requiring wound care, trauma patients, tumor patients, patients with intestinal obstruction, and those with peritonitis. Classically, all these diseases will trigger inflammation. This triggers the aggregation of inflammation in the endothelial system by ICAM-1 with transendothelial mechanisms that include attachment, activation, adhesion, and transmigration [24]. Furthermore, the above mechanism showed that there will be stimulation of the production of endothelial molecules that were involved in this transendothelial cascade process, included the adhesion molecule ICAM-1. Several things that were in line with this mechanism were leukocytosis, where there was an increase in the neutrophil segment and a neutrophil-lymphocyte ratio of 4.0. Thus, it was concluded that where there was inflammation, there was an increased production of ICAM-1 as an adhesion molecule.

In other clinical conditions, this is seen in laboratory tests where the average patient is anemic. This is not directly related to the occurrence of infection, except in a state of severe infection. The presence of a severe infection involves the mechanism of bone marrow suppression, where there will be a decrease in hemoglobin production, the emergence of kidney disorders, and reduced stimulation of erythropoietin alpha to hemoglobin production [25]. As previously described, patients infected with COVID-19 would experience coagulopathy, platelet aggregation disorders, and endothelial permeability disorders. In addition to this mechanism, it may also be related to the SARS-CoV-2 receptor on the ACE-2 receptor that affects vascular tone, so it could be related to one of the vascular responses to injury or trauma, namely vasoconstriction in the setting of vascular injury.

Furthermore, liver function biomarkers were found to be within normal limits, so they did not appear to have any effect on the patient population included in this study. There is an increase in function for the assessment of kidney function, but this is within mild limits. Some of the conditions that affect this condition are dehydration, impaired coagulability, and impaired vascular endothelium, which then affects the glomerular filtration ability of the kidney nephrons. The patient's hemodynamic condition can also affect kidney function, which will reduce the glomerular filtration rate so that kidney function can increase. In this situation, acute kidney injury can occur, however, if the patient is hydrated and hemodynamic, improvements are expected to occur [26]. In assessment of the change in the values of ICAM-1 levels during treatment, a decrease in ICAM-1 levels was seen during treatment at the time of initial patient care and when the patient's clinical improvement occurred. The clinical improvement that was observed correlates with reduced inflammation in the patient. This will be in line with the reduced stimulation of the ICAM-1 adhesion molecule, such that it is expected that ICAM-1 levels will be reduced. This was also determined by the degradation system of ICAM-1 which would eliminate ICAM-1 from the body. The results obtained showed a decrease, although this was statistically insignificant. This examination required further research conducted with a larger sample. Of all the samples examined, there was no significant increase in patients with confirmed COVID-19. This can be explained by the fact the SARS-CoV-2 receptors were not ICAM-1, unlike in other viral infections such as Rhinovirus whose cell receptor was ICAM-1, thus the subsequent inflammatory cascade after a COVID-19 infection could also increase the stimulation of the ICAM-1 adhesion molecule as shown and described previously. Such a phenomenon was not seen in this study.

## Conclusion

It can be concluded that the levels of ICAM-1 decreased in line with the clinical improvement of the patient. However, ICAM-1 levels were not significantly associated with COVID-19 infection yet were directly related to blood leukocyte levels. Larger-scale studies were needed to definitively establish the relationship between ICAM-1 levels and COVID-19 infections. In addition, further research on how ICAM-1 can act as a prognostic biomarker in surgical patients was needed to determine patient outcomes in the future.

### What is known about this topic

- *COVID-19 causes a systemic inflammatory response, which involves the dysregulation and incorrect expression of many inflammatory cytokines;*
- *Inflammatory cell recruitment and activation were dependent on the expression of many classes of inflammatory mediators, such as cytokines (interleukin [IL]-1, IL-6, and IL-18), chemokines (fractalkine [FKN]), and adhesion molecules (Intercellular Adhesion Molecule-1 [ICAM-1])*.

### What this study adds

- *ICAM-1 levels elevate in patients with COVID-19, dramatically so in severe cases, but decrease in the convalescent phase;*
- *ICAM-1 levels were not significantly associated with COVID-19 infection;*
- *ICAM-1 levels were significantly related to leukocyte levels*.
